# Expression of multidrug resistance markers ABCB1 (MDR-1/P-gp) and ABCC1 (MRP-1) in renal cell carcinoma

**DOI:** 10.1186/1471-2490-9-6

**Published:** 2009-06-24

**Authors:** Naomi Walsh, Annemarie Larkin, Susan Kennedy, Lisa Connolly, Jo Ballot, Wei Ooi, Giuseppe Gullo, John Crown, Martin Clynes, Lorraine O'Driscoll

**Affiliations:** 1 National Institute for Cellular Biotechnology, Dublin City University, Glasnevin, Dublin 9, Ireland; 2 St Vincent's University Hospital, Dublin 4, Ireland; 3 Current address - School of Pharmacy & Pharmaceutical Sciences, Trinity College Dublin, Dublin 2, Ireland

## Abstract

**Background:**

Renal cell carcinoma patients respond poorly to conventional chemotherapy, this unresponsiveness may be attributable to multidrug resistance (MDR). The mechanisms of MDR in renal cancer are not fully understood and the specific contribution of ABC transporter proteins which have been implicated in the chemoresistance of various cancers has not been fully defined in this disease.

**Methods:**

In this retrospective study the expression of two of these transporter efflux pumps, namely MDR-1 P-gp (ABCB1) and MRP-1 (ABCC1) were studied by immunohistochemistry in archival material from 95 renal cell carcinoma patients.

**Results:**

In the first study investigating MDR-1 P-gp *and *MRP-1 protein expression patterns in renal cell carcinoma patients, high levels of expression of both efflux pumps are observed with 100% of tumours studied showing MDR-1 P-gp *and *MRP-1 positivity.

**Conclusion:**

Although these findings do not prove a causal role, the high frequency of tumours expressing these efflux pumps suggests that they may be important contributors to the chemoresistance of this tumour type.

## Background

Kidney cancer accounts for approximately 2% of all adult cancers. It is the 7^th ^leading cause of cancer in the US with an estimated incidence of approximately 51,000 new cancer cases per year in 2007 [1]. Renal cell carcinoma (RCC) is the most common tumour arising from the cells in the lining of tubules in the kidney [2]. At the time of diagnosis, 30% of patients will have metastatic or unresectable disease, and the 2-year overall survival of this cohort is <10% [3]. The incidence of kidney cancer is rising; it is 2 times more common in men than women. Risk factors include obesity [4], smoking [5] and hypertension [6].

RCC is a chemoresistant tumour usually exhibiting only a marginal response. Radiotherapy and chemotherapy are generally ineffective in the treatment of advanced renal tumours [7,8]. The intrinsic occurrence of multidrug resistance (MDR) modulates the resistance of tumours to a wide variety of and structurally distinct chemotherapeutic drugs through the expression of drug efflux pumps [9]. Two of the most widely studied efflux pumps, MDR-1 P-gp/P-170, the gene product of MDR-1 (ABCB1) and MRP-1 (ABCC1) which encodes a 190 kDa membrane protein have both been demonstrated to pump a wide variety of the most commonly used cancer drugs out of tumour cells. Their over expression correlates broadly with drug resistance in many different forms of cancer including pancreatic cancer [10], lung cancer [11], breast cancer [12] and glioma [13]. The relative contributions and causative role, if any, of MDR associated protein efflux pumps in renal carcinoma have not been fully elucidated.

Studies detailing the prevalence and contribution of MDR-1 P-gp in RCC are conflicting. MDR-1 P-gp expression has been reported widely in untreated renal carcinomas [14,15]. It does appear that intrinsic drug resistance exists in many renal RCC and it is associated, at least in part, with increased expression of MDR-1 P-gp. However, the exact prognostic significance of this expression remains unclear with conflicting results described. Longer progression free survival has been observed in patients with none or very few MDR-1 P-gp positive tumour cells compared to patients with a larger proportion of MDR-1 Pgp positive tumour cells [16,17]; however higher MDR-1 expression has been associated with a better outcome also [18,19]. Expression of MDR-1 P-gp has been shown to correlate with a well differentiated tumour phenotype in renal carcinoma [18,20,21]. Higher MDR-1 gene expression has been observed in RCCs that have metastasised/invaded through the renal capsule compared to early stage non invasive tumours [20,22].

Studies addressing the contribution, if any, of the MRP-1 efflux pump and its gene product in this disease are limited. MRP-1, like MDR-1 P-gp is highly expressed in normal kidney. MRP-1 gene over expression has been observed in renal carcinomas, this expression does not appear to correlate with grade/clinical stage in this disease [19,23]. To our knowledge, there have been no reported studies looking at MRP-1 protein expression in RCC.

In this study, we evaluate the expression of MDR efflux pumps, MDR-1 P-gp and MRP-1, using immunhistochemical analysis, in 95 RCCs, to investigate the relative contributions of these efflux pumps in this disease.

## Methods

### Patients

The patient group consisted of 95 consenting patients diagnosed with primary renal cell carcinomas. All patients were treated at St. Vincent's University Hospital (SVUH), Dublin between 1999 and 2003. Approval to conduct this study was granted by the SVUH Ethics Committee. Pathological material was examined on each case by SK. Formalin-fixed paraffin-embedded material was available for all patients. Representative 4-μm sections of tissue blocks were cut using a microtome, mounted onto poly-l-lysine coated slides and dried overnight at 37°C. Slides were stored at room temperature until required. Clinicopathological features, where available were compiled for relevant patients.

### Immunohistochemistry

Immunohistochemical studies were performed on formalin fixed paraffin embedded renal carcinomas as described previously [12]; using anti-MDR-1 P-gp (antibody MDR-1, 6/1C; National Institute for Cellular Biotechnology [24]: ascites diluted 1:40) and anti-MRP-1 (antibody PA28(6), neat supernatant, National Institute for Cellular Biotechnology [25]). Positive control tissues (normal kidney and lung tissue) and negative control specimens in which primary antibody was replaced by 1XTBS/0.05% Tween 20 were included in all experiments.

### Immunohistochemical scoring

MDR-1 P-gp and MRP-1 immunohistochemical staining was evaluated semi-quantitatively, according to the percentage of cells showing specific immunoreactivity and the intensity of this immunoreactivity. Scoring involved evaluation of at least 5 fields of view per slide, by two independent observers. In the case of both MDR-1 P-gp and MRP-1, membrane *and *cytoplasmic staining was scored as positive or negative. A semi-quantitative measurement was used in which overall positivity of the tumour was assessed and a score of 1+ was given where up to 25% of cells showed MDR-1 P-gp/MRP-1 positive staining; a score of 2+ was given where ≥ 25% but < 50% of cells showed MDR-1 P-gp/MRP-1 positive staining; a score of 3+, where ≥ 50% but <75% of cells showed positive staining and a score of 4+, where ≥ 75% of cells showed positive staining. For assessment of both MDR-1 P-gp and MRP-1 protein, the intensity of immunoreactivity was scored as 1 (weak), 2 (moderate), or 3 (strong) as outlined in table 1.

**Table 1:** Percentage and intensity grade of staining.

**Percentage grade of staining**	**Intensity grade of staining **
1 = 1<25%	Level 1 = weak staining

2 = ≥25–50%	Level 2 = moderate staining

3 = ≥50<75%	Level 3 = strong staining

4 = ≥75<100%	

## Results

### MDR-1/P-gp expression

**Figure 1:**
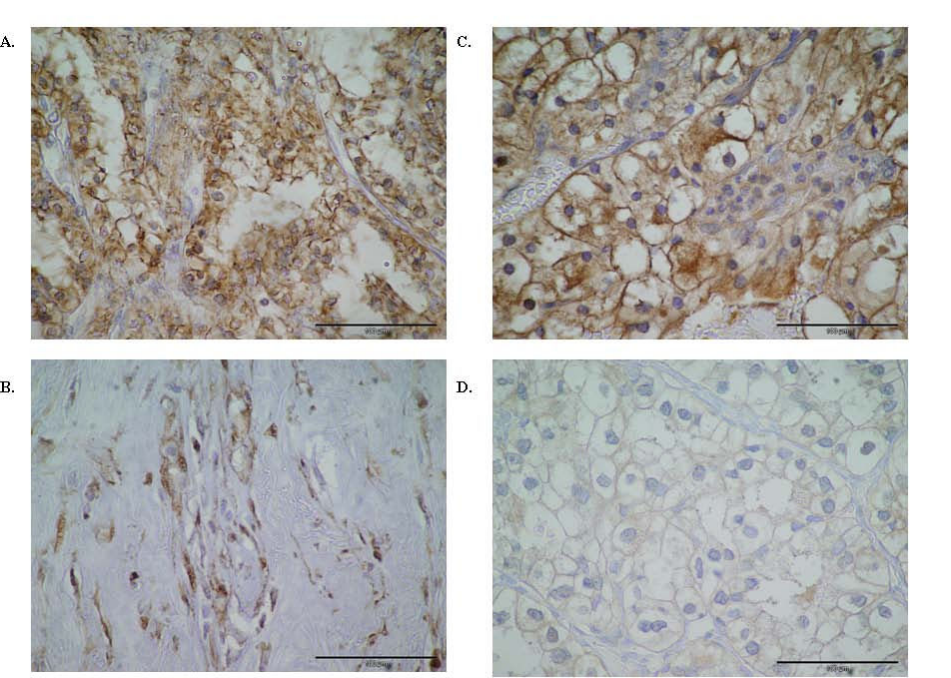
**Immunohistochemical analysis of MDR-1 P-gp and MRP-1 protein expression in RCC. **(A) RCC tumour showing positive MDR-1 P-gp staining with strong MDR-1 positivity observed, score (4+3) (scale bar = 100 μm) and (B) MDR-1 P-gp positive RCC with moderate positivity observed, score (2+2) (scale bar = 100 μm) (C) MRP-1 positive tumour showing intense MRP-1 positive staining, score (4+3) (scale bar = 100 μm) and (D) MRP-1 positive RCC with moderate positivity observed, score (2+2) (scale bar = 100 μm). (Original magnifications of all photomicrographs ×40).

The immunohistochemical analysis revealed that of the 95 cases, MDR-1 P-gp specific staining was observed weakly positive in 22% (21/95), moderate staining was observed in 40% (38/95) and strong staining in 38% (36/95) of RCCs analysed. Figure 1(A) shows a representative MDR-1 P-gp positive tumour where intense MDR-1 P-gp positivity is observed (score of 4+3) and (B) MDR-1 P-gp positive tumour with moderate staining intensity (score of 2+2). Specific staining was localised to the cell membrane and cytoplasm. The majority of tumours had between 50–75% positive staining for MDR-1 P-gp, associated with an intermediate (+2) intensity positive staining (3+2). 76% of tumours (72/95) showed MDR-1 P-gp staining in 50% or more of tumour cells. As outlined in Table 2, the distribution of MDR-1 P-gp expression was analysed by percentage staining, age, gender, tumour size, tumour grade and nodal status (if known). Of the 95 MDR-1 Pgp positive RCC, 6% (6/95) scored 1, 18% (17/95) scored 2, 45% (43/95) scored 3 and 31% (29/95) scored 4 percentage staining of MDR-1 Pg-p.

**Table 2:** P-gp expression in renal cell carcinoma and association with age at diagnosis, tumour size, histological grade and nodal status (*n *= 95)

	Number	Strong (+3)	Moderate (+2)	Weak (+1)
*Cases expressing P-gp*	95			
**Percentage Staining**				
1+ (1<25%)	6	0	2	4
2+ (≥ 25–50%)	17	5	8	4
3+ (≥ 50<75%)	43	9	23	11
4+ (≥ 75%)	29	22	5	2
**Age**				
< 60	41	20	13	8
> 60	54	16	25	13
**Gender**				
Male	49	26	17	6
Female	46	10	21	15
**Tumour size**				
< 7 cm	56	21	26	9
> 7 cm	39	15	12	12
**Tumour grade **(7 unknown)				
Grade 1	16	5	8	3
Grade 2	35	16	15	4
Grade 3	37	13	13	11
**Nodal status **(56 unknown)				
Positive	31	10	15	6
Negative	8	2	4	2

### MRP-1 expression

All 95 RCCs showed MRP-1 protein expression; MRP-1 specific staining was observed weakly positive in 21% (20/95), moderate staining was observed in 49% (47/95) and strong staining observed in 29% (27/95) of RCC analysed. Figure 1(C) illustrates an MRP-1 strongly positive (score of 4+3) tumour and (D) an MRP-1 positive tumour showing less intense MRP-1 staining (score of 2+2). Table 3 shows a breakdown of the distribution of MRP-1 positive tumours. Of the cases expressing MRP-1, 7% (7/95) scored 1, 32% (30/113) scored 2, 40% (38/95) scored 3 and 21% (20/95) scored 4. 61% of tumours (58/95) showed MRP-1 staining in 50% or more of tumour cells.

**Table 3:** MRP-1 expression in renal cell carcinoma and association with age at diagnosis, tumour size, histological grade and nodal status (*n *= 95)

	Number	Strong (+3)	Moderate (+2)	Weak (+1)
*Cases expressing MRP-1*	95			
**Percentage Staining**				
1+ (1< 25%)	7	0	2	5
2+ (≥ 25–50%)	30	2	16	12
3+ (≥ 50<75%)	38	13	23	2
4+ (≥ 75%)	20	13	6	1
**Age**				
< 60	41	14	19	8
> 60	54	14	28	12
**Gender**				
Male	49	15	25	9
Female	46	13	22	11
**Tumour size**				
< 7 cm	56	14	29	13
> 7 cm	39	14	17	7
**Tumour grade **(7 unknown)				
Grade 1	16	5	7	4
Grade 2	35	8	19	8
Grade 3	37	14	17	6
**Nodal status **(56 unknown)				
Positive	31	8	17	6
Negative	8	2	3	3

The highest percentage staining and intensity of MRP-1 expression was observed in male patients over the age of 60 with grade 2 tumours of < 7 cm in size and with positive nodal status.

### Correlation between MDR-1 P-gp and MRP-1

All tumours studied expressed both MDR-1 P-gp and MRP-1; intensity levels of these transporters did not vary to any great degree, however a higher proportion (76%) of MDR-1 positive tumours exhibited positive staining in 50% or more of tumour cells compared to MRP-1 staining patterns where 61% of MRP-1 positive tumours exhibited staining in 50% or more of tumour cells.

## Discussion

Chemotherapy is the standard treatment for most solid tumours; however, RCC is generally resistant to chemotherapy. The reason for the resistance of kidney cancer cells to chemotherapy is not completely understood. The specific role and relative contributions of ABC transporter pumps in clinical resistance in RCC have not been fully determined; results from previous studies are conflicting.

The MDR1 gene and its gene product, P-gp are ubiquitously expressed in mesangial, proximal tubule, thick loop of Henle, and collecting duct cells of the kidney [26]. As P-gp plays a functional role in the clearance of xenobiotics from the mesangial and proximal tubule cells of the kidney, this may contribute to the inherent multidrug resistance phenotype of RCC. MDR-1 gene overexpression or protein have been identified in the majority of RCC samples studied [14-17,19,21].

In this reterospective study we have shown that renal cancer cells produce an overabundance of this drug efflux pump, with 100% of RCCs studied exhibiting MDR-1 P-gp positivity. 76% of these tumours exhibited MDR-1 P-gp staining in 50% or more of tumour cells. As renal tissue inherently expresses high levels of this transporter; results presented here are not unexpected. Using a similar scoring system as that used here, Mignogna *et al*., [16] observed MDR-1 P-gp expression in 100% (30/30) of RCC. Other immunohistochemical studies on smaller patient cohorts report 61.5% (8/13) and 47.6% (10/21) of RCC showing MDR-1 P-gp positivity [14,15]; studies investigating MDR-1 gene expression in RCC report varying levels of gene expression, in general slightly less that the MDR-1 P-gp protein expression observed here [19-23]. It is well recognised that protein levels do not necessarily correlate with mRNA levels, furthermore these differences between the various studies may in part result from the different techniques employed and different cohorts of patients and scoring criteria used. The high levels of MDR-1 P-gp expression observed here do suggest that this transporter protein is contributing, at least in part, to the chemoresistance of RCCs studied here.

Mignogna *et al*., [16] suggested a role for MDR-1 P-gp as a possible adverse prognostic factor of chemoresistance and aggressive behaviour in renal carcinoma, their study showed an association between high MDR-1 P-gp expression (MDR-1 positivity in 40% or more of tumour cells) and poor survival as confirmed by Cox multivariate analysis. In agreement with this study, Duensing *et al*., [17] suggested a potential role for P-gp as a biologic parameter predictive of tumour progression in renal cell carcinoma patients, as longer disease-free survival was observed in patients with <1% MDR-1 postivity. However, Hofmockel *et al*., [18] correlated lower MDR-1 expression with poorer prognosis. Due to the lack of data regarding patient outcome; any possible prognostic significance of the expression of these efflux pumps observed could not be addressed in this study.

Expression of this efflux pump does not appear to be associated with the histological tumour grade of RCC in this patient cohort. Unexpectedly lower MDR-1 levels have been shown to be associated with poorly differentiated RCC [18,20-22]. However, in agreement with our observations Mignogna *et al*., showed MDR-1 protein expression as having prognostic significance independent of any association with tumour grade [16].

We have also shown high levels of MRP-1 protein expression in RCC; all tumours investigated showed MRP-1 protein expression with 61% of tumours exhibiting MRP-1 positivity in at least 50% of tumour cells. As in the case of MDR-1 Pgp, this efflux pump is also expressed at high levels in the normal kidney; so such an observed high level of expression again is not unexpected. This is the first report to our knowledge of MRP-1 protein expression being investigated in RCC patients, previous work has focused on gene expression studies. Again, this observed high level of MRP-1 protein expression suggests that this efflux pump also may be playing a contributing role in the chemoresistance of these renal carcinomas.

There was no apparent correlation between expression of either of the MDR proteins studied here with other clinicopathological features (gender, tumour size and nodal status). It was unfeasible to carry out detailed statistical analysis due to limitations and inadequate data. Histological grade, pathological stage and tumour size have been demonstrated using multivariate analysis to be significant prognostic indicators regarding RCC patient outcome [27]. Recently described molecular and genetic markers may also provide diagnostic and prognostic value in RCC [28].

It is likely that chemoresistance in RCC is multifactorial, other factors implicated as being involved in the complex chemoresistance mechanisms of RCC include *Clusterin *[29]; there also is some evidence that lung resistance protein (LRP/MVP) may contribute to inherently resistant renal carcinoma [30,31].

The inherent resistance of RCC to conventional treatment has lead to the use of immunotherapies such as alpha-interferon and interleukin-2 (IL-2). However, response does not translate to long-term benefit and does not prolong overall survival in many cases [32-34]. Newer "targeted-therapies" such as sunitinib [34,35] are emerging for the treatment of patients resistant/intolerant to current treatment modalities or as an alternative to cytokine immunotherapy. Identification of new tumour associated antigens may hopefully provide the basis of new therapeutic strategies and lead to an improved outcome for this aggressive and chemoresistant disease.

## Conclusion

Both MDR-1 P-gp and MRP-1 were expressed in 100% of the 95 specimens analysed. These high levels of expression suggest that both of these efflux pumps may be important contributors to the MDR phenotype in RCC. The incidence of MRP-1 positive tumours observed here warrants further study in order to confirm a possible contribution to chemoresistance in renal carcinoma. Due to the lack of data on the direct outcome of patients in this study, the prognostic significance of observed MDR protein expression was outside the scope of this investigation. Extensive studies with complete follow-up detailing expression of MDR-1 P-gp and MRP-1 together with further MDR associated markers (including LRP and MRP family members) and associated proteins may help to fully elucidate the specific contributions of these efflux pumps to the chemoresistance of RCC and furthermore address any possible prognostic and/or predictive role.

## Abbreviations

IL-2: interleukin-2; LRP: Lung resistance protein; MVB: major vault protein; MDR: Multiple Drug Resistance; MRP-1: Multiple Drug Resistance associated protein 1; MDR-1: Multiple Drug Resistance protein 1; RCC: renal cell carcinoma; SVUH; St. Vincents University Hospital; TBS: Tris buffered saline.

## Competing interests

The authors declare that they have no competing interests.

## Authors' contributions

LOD, MC & JC conceived of and designed the study. NW performed immunohistochemical studies and analysis and interpretation of results. AML contributed to design, interpretation of results and supervised the research. SK provided clinical material and scored slides. LC contributed to design of immunohistochemical studies. JB, WO & GG acquired clinico-pathological information. NW and AML drafted manuscript and revised it critically for important intellectual content. All authors read and approved the final manuscript.

## Pre-publication history

The pre-publication history for this paper can be accessed here:


